# Light‐Induced On‐Surface Reactions: Bridging Photochemistry and Surface Science

**DOI:** 10.1002/cplu.202500476

**Published:** 2025-09-19

**Authors:** Federico Frezza, Pavel Jelínek, Sofia Canola, Ana Sánchez‐Grande

**Affiliations:** ^1^ Department of surfaces and molecular structures Institute of Physics of the Czech Academy of Sciences Cukrovarnická 10 16200 Prague Czech Republic; ^2^ CATRIN—RCPTM Palacký University Olomouc Šlechtitelů 27 77146 Olomouc Czech Republic; ^3^ Organic Chemistry Department Faculty of Chemical Sciences Universidad Complutense Madrid Pl. de las Ciencias 2 28040 Madrid Spain

**Keywords:** molecular self‐assembly, on‐surface synthesis, photochemistry, photoreaction mechanisms, scanning tunneling microscopy

## Abstract

Molecular on‐surface photochemistry recently emerged as an alternative strategy to thermal reactions to synthesize low‐dimensional carbon‐based nanomaterials, particularly on nonmetallic surfaces. However, there is still limited knowledge about the crucial aspects influencing photoreactivity in the context of surface chemistry, which contrasts with the fast progress in thermally activated reactions on metal surfaces. By reviewing recent developments in the on‐surface photochemistry field, this minireview focuses on some key aspects crucial for the comprehension of photoreactions on surfaces: from the photoexcitation process to basic mechanistic aspects and intermediates characterization, including the molecular preorganization impact on the reaction evolution. To clarify these aspects, we rely upon well‐established concepts of traditional photochemistry while highlighting the role of the surface. Finally, it is concluded with considerations on the evolution of the field.

## Graphical abstract

This minireview examines molecular on‐surface photochemistry as an emerging strategy for synthesizing carbon‐based nanomaterials on substrates. A special emphasis is placed on bridging traditional photochemistry principles with surface science concepts. We discuss photoexcitation mechanisms under ultra‐high vacuum conditions, reaction pathways following light activation, and the critical role of molecular preorganization in determining reaction outcomes.

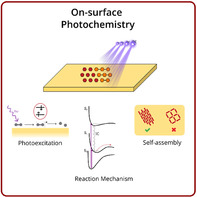

## 1 Introduction

In the last decades, the potential of on‐surface chemistry to synthesize carbon‐based nanostructures has been demonstrated.^[^ ^1^ ^,^ ^2^ ^]^ Most reactions in the context of on‐surface synthesis (OSS) take place under ultra‐high vacuum conditions (UHV) and are thermally activated reactions on metal substrates, where the catalytic role of the surface plays a pivotal role.^[^ ^3^, ^4^ ^–^ ^5^ ^]^ In parallel, the investigation of light‐induced reactions on surfaces was gaining importance, not only from the point of view of expanding the toolbox of reactions on surfaces, but also because photochemical reactions do not need the catalytic metal substrate. This may facilitate the pathway to the ultimate goal: the synthesis of carbon‐based nanostructures presenting applications in nanodevices.^[^ ^6^ ^,^ ^7^ ^]^ The main limitation to integrating the molecular systems into nanodevices is the transfer process from a metal surface to a technologically relevant substrate.^[^ ^8^ ^]^ In this context, photochemical reactions offer the opportunity of synthesizing these carbon‐based nanomaterials directly on the desired substrate, avoiding transfer procedures. Another advantage associated with photochemical reactions is the plausible “prediction” of the reaction outcomes based on the optical properties of the molecular systems. In many cases, thermal reaction products present abundant defects, associated with simultaneous activation barriers of different reaction pathways. Thus, photochemical reactions present the opportunity of designing highly selective reactions on different substrates, including semiconducting and insulating surfaces.

Photochemistry is a well‐established field that started by the end of the 19th century, with pioneering works from Ciamician and Silber.^[^ ^9^ ^]^ Major advances in the study of photochemical reactions occurred in the 20th century,^[^ ^10^ ^]^ leading to several novel reactions. In parallel to experimental development, interpretative models based on the molecular electronic structure have been developed to qualitatively characterize photoreaction mechanisms and offer predictions on the photoproducts.^[^ ^11^, ^12^ ^–^ ^13^ ^]^ Now, the tools of computational quantum chemistry permit a complete characterization of organic photoreaction mechanisms for molecules of small to medium dimension.^[^ ^14^, ^15^ ^–^ ^16^ ^]^ From this point of view, the knowledge developed in the photochemistry field could be transferred to surface photochemistry, taking inspiration from well‐known reactions.^[^ ^17^ ^]^ However, the surface is not completely innocent, and it might play a very important role in the reaction course, such as in the light absorption process or the adsorption geometry of the molecules.

In this minireview, we aim to present the primary aspects to consider in the framework of on‐surface photochemistry involving covalent bond formation/dissociation, mainly focusing on reactions under UHV conditions. We highlight some important aspects about the role of the surface but also describe some analogies with traditional photochemistry. The idea is to gather important considerations in the context of light‐mediated reactions on surfaces and contribute to bridging the gap between photochemistry and surface science, rather than an exhaustive review of the literature. For the latter purpose, we refer the readers to previous review works.^[^ ^18^, ^19^, ^20^, ^21^ ^–^ ^22^ ^]^ This minireview consists of four different sections: 1) in the first section, we consider reactions taking place under UHV conditions, focusing on the mechanism behind the photoexcitation process, how the surface could propose alternative mechanisms and how the optical properties of the molecular system could be affected by the molecule‐surface electronic interactions. 2) The second section aims to understand the reaction mechanism following photoexcitation, still sticking to works performed under UHV conditions. 3) The third section shows a more extensive review of works performed both under UHV conditions or at solid/liquid and solid/air interfaces, highlighting the importance of the molecular self‐assembly and drawing some analogies with the topochemical postulates and the photochemistry of organic solids. 4) The last section highlights the potential of surface photochemistry for the study of relevant highly reactive reaction intermediates. Altogether, we aim to contribute to establishing a systematic understanding and development of the field of photochemistry on surfaces and motivate the use of technologically relevant substrates for the course of the reactions.

## 2 Electronic Interactions and Photoexcitation Mechanism

This section focuses on the role played by the surface in the photoexcitation of an adsorbed molecule and how it affects its photophysical properties, examining the excitation and relaxation processes occurring just after photon absorption. Here, we will discuss the possible processes leading to a transient state, which subsequently initiates a chemical reaction under the following assumptions: 1) an organic molecule is adsorbed on a surface in the physisorption regime, i.e., the interaction energy is weak. However, a renormalization of the molecular electron density due to interactions with the substrate can occur, which, in most cases, is reflected in a reduction of the gap due to polarization effects and the broadening of the molecular levels.^[^ ^23^ ^]^ 2) The molecule‐substrate system is in vacuum. Those approximations are valid for most of the systems reported in this review, where organic molecules are weakly adsorbed on metallic or semiconducting/insulating surfaces, and the experiments are carried out in UHV conditions, with few notable exceptions at the air/solid or liquid/solid interface.

**Scheme 1:**
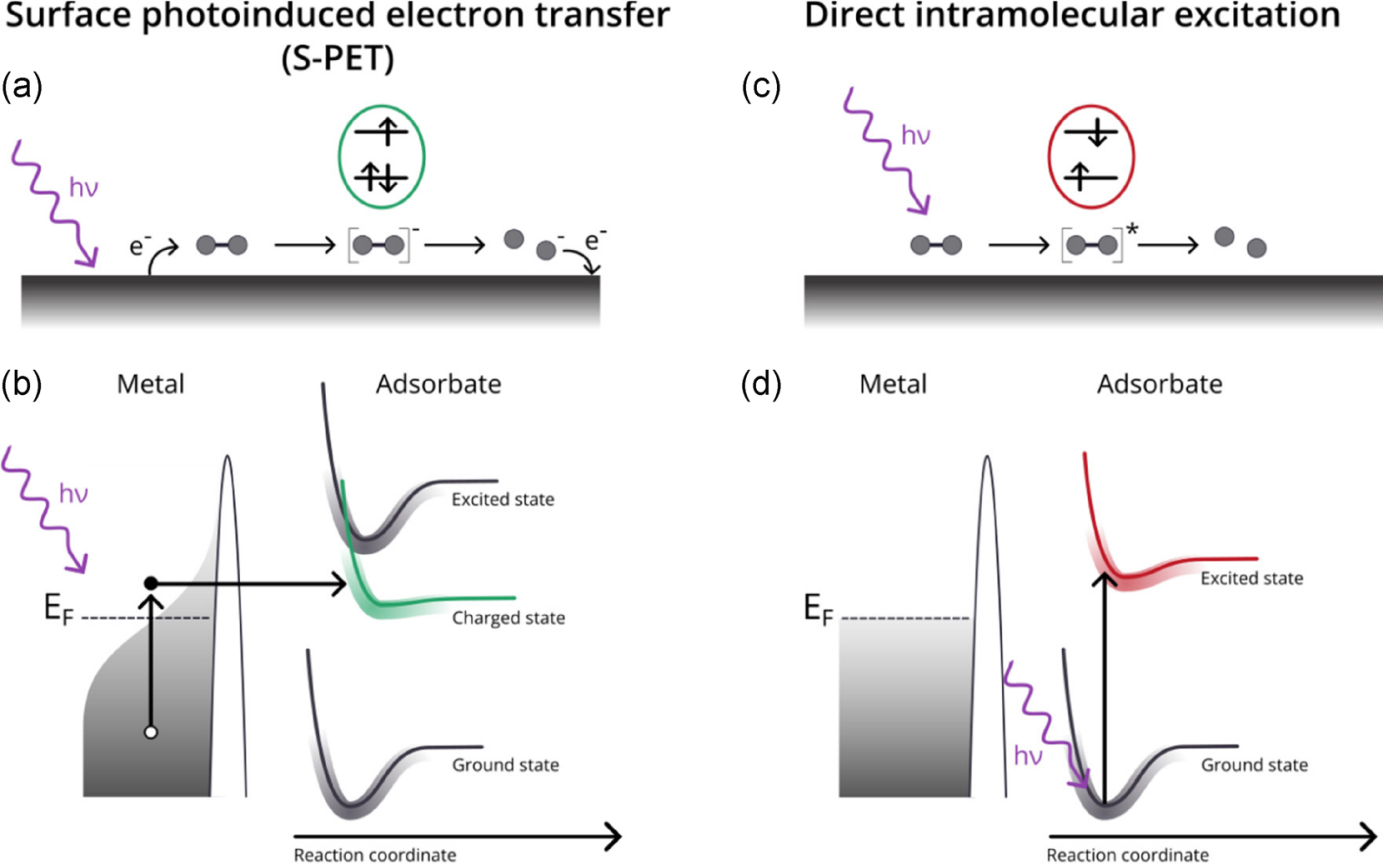
Two general mechanisms for light‐induced chemistry of adsorbates. The sketch represents the simple case of photodissociation on a metal surface. In the bottom panels the adsorbate potential energy curves are blurred to depict the level broadening occurring on surfaces. a,b) S‐PET, where a photogenerated hot electron from the substrate can be attached to the adsorbate leading to an active anionic state (green curve). c,d) Direct intramolecular excitation following the absorption of a photon from the molecule. The adsorbate initiates the reaction from an excited state.

Under these conditions, two main mechanisms can lead the adsorbed species to an active state, which precedes a chemical reaction (**Scheme** **1**): surface‐photoinduced electron transfer (S‐PET) and direct intramolecular excitation.^[^ ^24^ ^,^ ^25^ ^]^

In the case of the S‐PET mechanism, the absorption of photons by the metal generates holes in the valence band and “hot” carriers above the Fermi energy (E_F_) that can be injected into unoccupied states of the organic adsorbate (Scheme 1a). This generates an anionic state (green in Scheme 1a,b), which acts as an intermediate to a chemical transformation.^[^ ^26^ ^,^ ^27^ ^]^ In literature, different terms such as “hot electron attachment”, “dissociative electron attachment”, “charge–transfer photodissociation” “photoinduced charge transfer”, and “substrate excitation”, are also used to identify the processes where hot carriers are transferred to the adsorbate. We decided to adopt the nomenclature of "surface photoinduced electron transfer” (S‐PET), in analogy with the commonly described PET between donor and acceptor molecules.^[^ ^28^ ^]^ This process requires the participation of surface electrons, which makes it the predominant mechanism in the case of metal surfaces.

S‐PET was proposed to be the predominant mechanism in a series of early works on the light‐induced dissociation of small molecules on metal surfaces such as HCl on Ag(111),^[^ ^29^ ^]^ O_2_ on Ag(110),^[^ ^30^ ^]^ NO_2_ on Pd(111),^[^ ^31^ ^]^ and more. In a study of CH_3_Cl dissociation on Ni(111),^[^ ^32^ ^]^ the authors were able to recognize the fragmentation following S‐PET by changing the irradiation wavelength and detecting the photoproducts with time‐of‐flight techniques. In particular, the dissociation observed after illumination at 248 nm is explained by S‐PET from the underlying metal. For higher molecular coverages (>10 ML) no CH_3_ fragments are detected (the reaction is not happening in the higher layers), since the substrate's hot electrons can no longer be attached to the molecular top layer. In 1991, the first direct proof of the presence of an anionic state triggering the reaction was reported, detecting negative ions desorbing from a CCl_4_ film on Ag(111) surface after UV laser irradiation.^[^ ^33^ ^]^ This provides clear evidence that S‐PET is the underlying mechanism for CCl_4_ dissociation: the negative molecular ion dissociates to produce one neutral (CCl_3_) and one anionic fragment (Cl^−^). The latter one desorbs and can be detected by time‐of‐flight mass spectrometry, even in the absence of photoelectron emission (i.e., when the photon energy hv is lower than the substrate work function *ϕ*).^[^ ^33^ ^]^

(1) $$e^{-}+{\text{CCl}}_{4}\to {{\text{CCl}}_{4}}^{-}\to {\text{CCl}}_{3}+{\text{Cl}}^{-}$$

Another technique that allows for addressing the interfacial electronic states involved in the S‐PET is two‐photon photoelectron spectroscopy (2PPE), which has been successfully employed in surface photochemistry studies.^[^ ^28^ ^,^ ^34^ ^]^ 2PPE is a pump‐probe technique that can identify the electronic states involved in the photoreaction, rather than characterizing the photoproducts. The transient anionic state responsible for the phenol dissociation on Ag(111) has been identified:^[^ ^35^ ^]^ an unoccupied intermediate state was found 3.22 eV above the Fermi level, in agreement with the photodissociation threshold. This molecular resonance state, localized on the adsorbate, is generated by S‐PET, leading to molecular dissociation. Supporting the indirect excitation mechanism, the polarization dependance of the 2PPE shows that photoemission intensity scales with the absorbance of the substrate. The above‐mentioned photodissociation of CCl_4_ on Ag(111) was also studied by the same group by real‐time 2PPE, revealing a CCl_4_‐derived unoccupied image potential state at 3.41 eV above the Fermi level, with its transient population facilitating electron transfer to form the dissociative CCl_4_^−^ anion intermediate.^[^ ^36^ ^]^

**Figure 1:**
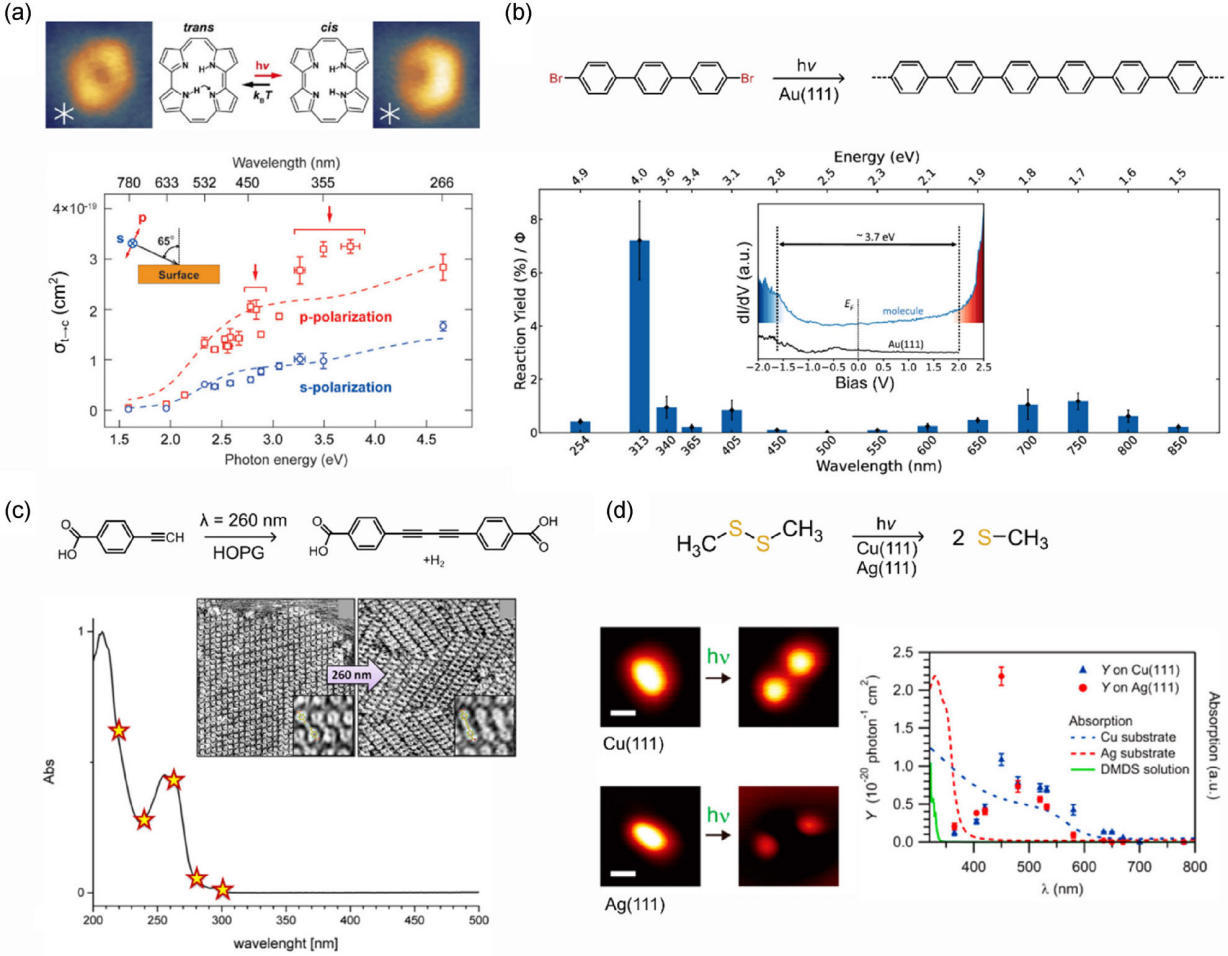
a) Chemical sketch and STM images of a porphycene molecule on Cu(111) in *trans* and *cis* configuration. The graph displays the photon energy (wavelength) dependance of the *trans–* *cis* tautomerization cross section (*σ*_t→c_), measured with p‐ and s‐ polarized light. Reproduced with permission.^[^ ^38^ ^]^ Copyright (2016), ACS. b) Reaction yields at different wavelengths for the light‐induced debrominated coupling on Au(111) of DBTP precursor. The inset shows STS spectra acquired on the intact molecule and on Au(111), showing an electronic gap of 3.7 eV. Reproduced with permission.^[^ ^40^ ^]^ Copyright (2025), ACS. c) Chemical sketch and STM images before and after irradiation of the photochemical homocoupling of terminal alkynes on HOPG. The absorption spectrum of the precursor in methanol is displayed, and the stars correspond to the excitation wavelengths employed by the authors. Reproduced with permission.^[^ ^43^ ^]^ Copyright (2016), ACS. d) Chemical sketch of the (CH_3_S)_2_ photodissociation and STM images before and after irradiation at 532 nm on Cu(111) and Ag(111). The graph shows the experimental photodissociation yield (Y) as a function of the wavelength. Blue and red dotted lines are the simulated absorption spectra of bulk Cu and Ag, respectively. The green line is the absorption spectrum of a (CH_3_S)_2_ solution. Reproduced with permission.^[^ ^44^ ^]^ Copyright (2017), ACS.

In the last 20 years, scanning tunneling microscopy (STM) and noncontact atomic force microscopy (nc‐AFM), have become the technique of choice to characterize molecular structures at surfaces and follow chemical transformation at the nanoscale with unprecedented spatial resolution. Nevertheless, STM allows for a detailed characterization of the products but makes it challenging to get direct information on the photoexcitation mechanism, despite the progress in the scanning tunneling microscopy‐induced luminescence (STML) field.^[^ ^37^ ^]^ Thus, to gain insights into the role of the surface in the excitation, it is necessary to change external parameters, such as the substrate, light wavelength, or polarization. This approach was used in the study of the light‐induced cis‐trans tautomerization of porphycene on Cu(111) (**Figure** **1**a).^[^ ^38^ ^]^ For both s‐ and p‐polarized light, the authors observe a steep increase of the tautomerization yield for a photon energy around 2 eV, matching the edge of the Cu (copper) d‐band, which is explained by an indirect S‐PET mechanism. By monitoring the photoreaction yield (defined as the number of reacted molecules divided by the total number of molecules) as a function of the light polarization for a certain wavelength, S‐PET was found to be the main excitation mechanism at 405 nm, also in the case of debromination of 4,4″‐dibromo‐p‐terphenyl (DBTP) on Au(111).^[^ ^39^ ^]^ The Au (gold) absorbance at 405 nm at different incident angles is distinctly different for p‐ and s‐polarized light. The substrate photoabsorption can approximate the reaction probability in the case of S‐PET. The reaction yield as a function of the irradiation angle matches the absorbance of gold, pointing toward the S‐PET mechanism. The same system was further investigated, monitoring the yield on a wide range of wavelengths, from UV to near infrared (Figure 1b).^[^ ^40^ ^]^ The trend is not linear, and the authors assign it to three distinct excitation mechanisms: charge transfer from the Fermi level of the metal substrate to the LUMO (700–750 nm), surface photoinduced electron transfer (405 nm), and direct intramolecular excitation (313 nm, highest efficiency).

Direct intramolecular excitation is based on the resonant intramolecular excitation forming an electron–hole pair after photon absorption (red in Scheme 1c,d). In contrast with the S‐PET mechanism, here the excitation is purely intramolecular, and the charge state of the molecule is preserved upon photon absorption (note that in most cases, organic molecules are neutral on surfaces, resulting in a neutral excited state). Thus, in principle, the molecular behavior upon irradiation presents more analogies with excitations in the gas‐phase or solution, even though the surface can induce distinct renormalization of molecular electronic states and consequently alter the excitation energies with respect to gas‐phase/solution. Furthermore, while excitation quenching and substrate electron attachment are hindered on nonmetallic surfaces, photochemical processes are often in competition with photodesorption of the molecules due to the low adsorption energies.^[^ ^22^ ^]^

In surface photochemistry, the direct excitation mechanism is easily identified in the case of reactions on large bandgap insulators, which are transparent to UV/Vis radiation, the energy range of most photochemical reactions. Few representative examples of this scenario are provided by early works on dehalogenation reactions on insulators, such as chloromethane on LiF(001)^[^ ^41^ ^]^ or on MgO, or iodobenzene on sapphire(0001).^[^ ^42^ ^]^ In these cases, the observed photoreactions are proposed to follow a direct intramolecular excitation, only taking place within the molecule. More recently, the photodimerization of terminal alkynes on graphene was studied by STM and wavelength‐dependent experiments (Figure 1c).^[^ ^43^ ^]^ By measuring the number of reacted molecules while changing the irradiation wavelength, the maximum photoconversion was found to match the absorption maximum of the precursor, consistent with a direct photoexcitation mechanism. As stated before, the presence of the surface can strongly influence the reaction conditions even in the case of direct molecular excitation. It was demonstrated that S—S bond dissociation in a (CH_3_S)_2_ molecule on Ag(111) and Cu(111), see Figure 1d, proceeds in the visible wavelength range, which is largely outside the absorption window of the compound.^[^ ^44^ ^]^ However, the authors do not assign this behavior to a substrate‐mediated excitation process but rather to a strong molecule substrate hybridization, reducing the HOMO—LUMO gap and altering the character of the frontier orbitals. The LUMO‐derived molecular orbitals have negligible overlap with the metal substrate, resulting in longer lifetimes of the excited state. In this case, the authors carry out the same photoreaction on different metallic surfaces, studying the reaction yield as a function of the wavelength (Figure 1d), observing how the curve does not follow the simulated photoabsorption spectrum of the Cu and Ag substrates, concluding that the course of the reaction is not strongly influenced by the type of surface.

We must emphasize that S‐PET and direct intramolecular excitation processes can and will compete in most cases, with different probabilities and cross‐sections depending on the system and the timescales of the different processes. It must also be noted that not all the photoexcited substrate electrons take part in the S‐PET. There is indeed a third scenario: some light‐generated hot carriers may not reach the surface or not be attached to the adsorbate (or photoemitted). Since the UV photon penetration depth in most metals is in the order of 100–1000 nm and scattering lengths are slightly shorter, a consistent number of the carriers generated will undergo inelastic scattering before reaching the surface. Their energy will be dissipated in the solid as heat. Thus, thermal activation can trigger (ground‐state) chemical reactions. Distinguishing this process from true photochemical excitation is crucial when interpreting reaction yields and mechanisms: for thermally‐driven processes, reaction rates depend solely on the total energy absorbed by the substrate, with no specific photon energy requirements beyond basic metal absorption.^[^ ^24^ ^]^ Thus, experimental distinction between these mechanisms can be achieved through wavelength‐dependent studies to identify photon energy thresholds, low‐temperature experiments where thermal pathways are suppressed, or by monitoring the reactivity on nonmetallic surfaces.

In summary, metal surfaces can play a dual role in on‐surface photon‐mediated chemistry. On the one hand, they can extend molecular reactivity to longer wavelengths by substrate‐molecule hybridization or by enabling substrate‐mediated excitation through S‐PET, where the reaction happens through a negatively charged transition state. On the other hand, they often promote ultrafast quenching, which suppresses the reactivity of excited states and limits the overall efficiency. In contrast, photochemistry on insulators more closely resembles gas‐phase behavior, where direct molecular excitation dominates and charge transfer and quenching effects are minimal. Semiconductors represent an intermediate case where bandgap properties may enable wavelength‐selective photocatalysis, with better charge separation reducing recombination rates and extending excited state lifetimes compared to metals.^[^ ^24^ ^,^ ^45^ ^]^ Understanding and controlling these competing effects is key to designing efficient surface photochemical systems and understanding their mechanism.

## 3 Photoreaction Mechanisms, Selectivity, and Yield

As described in the previous section, the outcome of the excitation process is either to drive the molecule into its excited state via direct excitation or to charge the molecule into an anionic state via S‐PET. For a reaction to occur, the states must be long‐lived enough, which means that competing quenching processes (de‐excitation or charge transfer to the surface) must be less efficient than the reaction. In the S‐PET case, a reaction is initiated and evolves on an ionic electronic state, possibly the lowest (ground) one, obtained by population of a virtual molecular orbital. Often, such states have a dissociative character and lead to bond breaking. This kind of reactivity can be referred to as photon mediated chemistry or photoelectron chemistry.^[^ ^28^ ^]^

**Figure 2:**
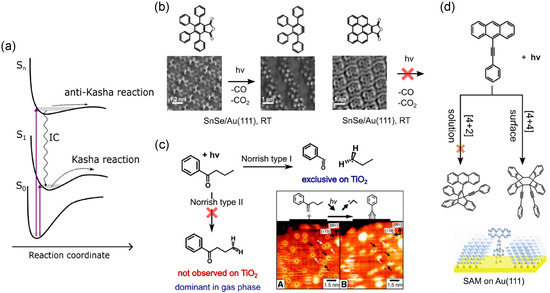
a) General scheme of potential energy curves of a molecule, along a reaction coordinate. Reproduced with permission.^[^ ^100^ ^]^ Copyright (1996), ACS. b) Anhydride photolysis of derivative TPPA and BPA on SnSe: nc‐AFM images before and after irradiation at 280 nm, RT. Reproduced with permission.^[^ ^47^ ^]^ Copyright (2024), Wiley. c) Photocleavage (Norrish type) of butyrophenone on TiO_2_: reaction paths sketch and STM image before and after irradiation with Xe lamp. Reproduced with permission.^[^ ^50^ ^]^ Copyright (2013), RSC. d) [4 + 4] cycloaddition of 9‐(4‐mercaptophenylethynyl)‐ anthracene on Au(111): reaction paths and SAM sketch. Reproduced with permission.^[^ ^54^ ^]^ Copyright (2011), AAAS.

In this section, we focus only on the case of the direct intramolecular excitation process, where a molecule adsorbed on the surface is promoted to an excited state via intramolecular excitation, and the reaction course proceeds from it (photochemistry) (**Figure** **2**a). Usually it occurs on insulating surfaces, as they are transparent at the typically employed wavelengths (UV–vis) and the molecules are physisorbed. The mechanistic interpretation can then be based on the molecular electronic properties, assuming that they are not dramatically altered from the ones in the gas phase, adapting the concepts developed for photochemistry in solution.^[^ ^46^ ^]^

In general, addressing the photoreaction mechanism in the presence of a surface is a complex task both from an experimental and a theoretical point of view, that so far has been discussed in a limited number of studies.^[^ ^47^, ^48^, ^49^ ^–^ ^50^ ^]^ Scanning probe microscopies can provide some indirect mechanistic information thanks to their excellent spatial resolution, for example, characterizing products and intermediates when stabilized on a surface (more in Section 5). However, STM time resolution is so far limited and makes it very difficult to track any processes below seconds (imaging)^[^ ^51^ ^]^ or ns (local spectroscopy), hampering the possibility to follow the reaction in time with sufficient time resolution. Theoretical modeling of photoreaction mechanisms, including the effect of the substrate in quantum chemical calculations, is very complex and has not been investigated in depth yet. Modeling of photoreactions requires a precise description of the molecular electronic structure in an excited state and its evolution along the reaction coordinate. Large molecular systems, including the surface, are typically treated with simpler methods (usually single‐determinant density functional theory).^[^ ^14^ ^,^ ^52^ ^,^ ^53^ ^]^ A careful description of photoreactivity would require more accurate methods (multireference), which are usually not computationally tractable for extended molecular systems.^[^ ^15^ ^,^ ^16^ ^]^

The evolution on the potential energy surface of an excited state follows a generic scheme similar to that sketched in Figure 2a. During the excitation, molecules can reach higher excited states, based on the energy of the promoting stimulus and the accessibility of states (selection rules). A general assumption is that the photoexcited molecules then relax to the lowest excited state (S_1_) via internal conversion (IC), which is usually assumed to be a fast process (Kasha's rule, Figure 2a).^[^ ^24^ ^]^ Once in S_1_, the photoreaction takes place, so that the reaction yield is independent of the excitation wavelength, provided that the irradiation energy is larger than the energy of S_1_. In general, once the lowest excited state is reached, the photoreaction mechanism will depend on its electronic characteristics and how it evolves along the reaction coordinate. The molecular structure of the precursor determines the electronic properties, and modifications of its structure impact the energy, the order and the electronic characteristics of the excited states. It was demonstrated for the photoreactivity of different derivatives of maleic anhydride on SnSe (Figure 2b):^[^ ^47^ ^]^ C—O bond dissociation occurs on a dissociative excited state with nπ* character, namely involving an excitation promoted from an orbital centered on the O lone pairs. The photoreactivity was tuned by structural modifications of the precursor: for the smaller tetraphenyl phthalic anhydride (TPPA) molecule, the reaction occurs efficiently since the reactive state is the lowest one, accessible by photoexcitation. When the π‐conjugated backbone was expanded to a benzoperylene (BPA) molecule (Figure 2b), the reactivity was suppressed: this has been attributed to the appearance of nondissociative ππ* states energetically lower than the nπ* one.^[^ ^47^ ^]^ In some cases, e.g. in the presence of heavy atoms such as halogens, the system can undergo intersystem crossing (ISC) to a potential energy surface of different spin multiplicity on which the reaction path evolves. This has been proposed for the debromination of a dianthracene derivative on mica, which evolves on the lowest triplet states after ISC from singlet excited states.^[^ ^48^ ^]^ Reactivity on higher energy excited states (antiKasha mechanism) can be feasible when the molecule has accessible ultrafast paths starting from higher excited states or when interstate relaxation mechanisms are hindered. This has been suggested for N—N breaking of anthraquinone‐diazide physisorbed on Au, that follows an ultrafast dissociative path originating from the state directly accessed by light (higher than S_1_).^[^ ^49^ ^]^

The substrate presence can directly affect the photochemical mechanism by influencing the energy of the excited states. Consequently, their accessibility can be limited for the available wavelengths, or their order can be altered with respect to gas/solution phase, leading to unwanted products (opening different reaction paths) or hindering the reaction (pushing reactive states higher in energy). In this regard, the surface can represent a limiting factor. Concretely, this can happen by affecting the molecular adsorption geometry, i.e., by constraining it far from gas or solution phase one, or by acting on the electronic structure, e.g., affecting orbital energies (orbital renormalization, as discussed in Section 2), although to a limited extent when physisorbed on semiconductors. There are also indirect ways in which the surface can influence the mechanism: it can modify the ground state electronic structure by interacting with adatoms or defects, potentially modulating the barriers; adatoms or defects can immobilize the precursors in different states or trap photoproducts or elusive intermediates.^[^ ^50^ ^]^ Because of a combination of these indirect effects, a mechanistic change (“selectivity switch”) was observed for the C—O cleavage of butyrophenone (asymmetric ketone) on the oxidized TiO_2_ rutile surface (Figure 2c): the photoreaction mechanism is altered due to the interaction with O adatoms (Norrish type II mechanism) with respect to the one commonly observed in gas phase (Norrish type I).^[^ ^50^ ^]^ The surface can also affect the reaction mechanism by directing the packing of molecules and creating self‐assembly that influences the reaction course (more on this in Section 4). As an example, the cycloaddition reaction of ethynyl‐phenyl substituted anthracene undergoes a mechanistic change when organized in a self‐assembled monolayer (SAM) (Figure 2d).^[^ ^54^ ^]^ In solution, the most probable reaction is [4 + 2] addition between anthracene central C atoms (9,10 position) and the alkyne moiety (triple bond). Inserting the molecule in a SAM (Figure 2d) forces anthracene molecules in an upright fronting position, by means of a rigid spacer with S termination interacting with Au surface. In this way, prearranging the molecules in π—π interacting right position and limiting their mobility, the reaction was forced in the less favorable [4 + 4] mechanism. In this case, it is mainly due to the steric hindrance originated by the geometric organization imposed by the surface, an effect which will be further elaborated in Section 4.^[^ ^54^ ^]^

In many cases, photochemical reactions on surfaces are more selective than thermal‐induced reactions: for the latter, activation barriers with similar energies might open bifurcations in the reaction pathways, resulting in side products and the presence of defects. Photochemical reactions have been demonstrated to provide higher control over the reaction evolution and result in highly selective products. This was, for example, demonstrated for the dehalogenation and subsequent Ullman coupling of dibromo biphenyl on Cu(111): the thermal activation results in various products while the photolytic reaction delivers only one product and has, in general, higher selectivity.^[^ ^55^ ^]^ The photoinduced deoxygenation of a dibenzothiophene sulfoxide derivative on NaCl showed a higher efficiency and selectivity with respect to the analogous one performed in dichloromethane solution.^[^ ^56^ ^]^ The interaction with the surface and the geometric organization of the precursors can make the reaction stereoselective: it is the case of porphyrin derivatives coupling on Ag(110) via N—H bond photodissociation^[^ ^57^ ^]^ or the dimerization of ester‐carbenes on Ag(111), where the less thermodynamically stable *cis* product is favored^[^ ^58^ ^]^ (more on this in Section 5).

Summarizing, photoreactions on surfaces have been shown to be generally efficient and selective, although with results dependent on the surface nature^[^ ^58^, ^59^, ^60^ ^–^ ^61^ ^]^ or the design of the molecular precursor.^[^ ^48^ ^]^

## 4 Impact of Molecular Preorganization

When molecular precursors are deposited on a surface, they can spontaneously self‐assemble, forming a 2D organization driven by molecule–molecule and/or molecule–surface interactions. This 2D organization of organic molecules forms a condensed phase that is crucial for the understanding of the reaction evolution. In this regard, we can draw similarities between photochemistry within the self‐assembly of molecules on surfaces and organic solid‐state photochemistry. In the 20th century, the investigation of molecular photochemistry in solid state gained importance thanks to the understanding of viable reactions or stereoselective reactions, based on topochemical postulates. Topochemistry defines the “geometrical” conditions/restrictions needed for a reaction to take place in the solid state.^[^ ^62^ ^,^ ^63^ ^]^ Topochemistry and photochemistry of organic solids are two fields closely related. However, it is particularly important to emphasize that these postulates have certain limitations,^[^ ^64^ ^]^ since molecular movements could depend on reaction conditions (e.g air‐solid vs. liquid–solid), the distortion of the lattice in unreacted molecules adjacent to product molecules that do not fit in the lattice, or dynamics in the excited state, as exemplified in some works throughout this section. Nevertheless, they have been demonstrated to explain and anticipate reaction outcomes within organic solid crystals. In this section, we aim to highlight analogies of “traditional” photochemistry of organic solids with molecular photochemistry on surfaces, including both reactions taking place under UHV conditions or at the solid–liquid/solid–air interface. When convenient, we will relate the self‐assembly packing on the surface to the topochemical postulates, referring to some works where the preorganization of the molecules follows the topochemical postulates,^[^ ^62^ ^,^ ^63^ ^,^ ^65^ ^]^ promoting coupling reactions with minimum molecular movement.^[^ ^66^ ^]^

In the context of photochemical reactions on surfaces, the molecular packing of the precursors can have a direct impact on the reaction evolution. In those cases where the precursors movement is confined, a favorable prearrangement (distance, orientation) of the reacting species is crucial. Contrary to thermal‐induced reactions, we cannot assume that molecular diffusion initiates intermolecular reactions. This has been evidenced by comparing the same reaction initiated by heat and light, in both cases inducing the formation of the same polymers but with significantly different lengths.^[^ ^67^ ^]^ The limited diffusion of the monomers at room temperature (RT) results in the formation of short oligomers coexisting with intact monomers upon UV irradiation, while at 77 K the photodebromination reaction is induced, but the monomers are found isolated. Light‐assisted thermal reactions can be an alternative to induce molecular diffusion upon light activation (C—X bond breaking)^[^ ^68^ ^]^ or to reduce activation barriers.^[^ ^69^ ^]^ Photochemical reactions are valuable because they can take place at low (cryogenic) temperatures, allowing the study of highly reactive compounds without the involvement of diffusion (mobility of species is suppressed). Therefore, to achieve efficient photochemical reactions on surfaces at low temperatures, in many cases, we must consider the topochemical factor.

**Figure 3:**
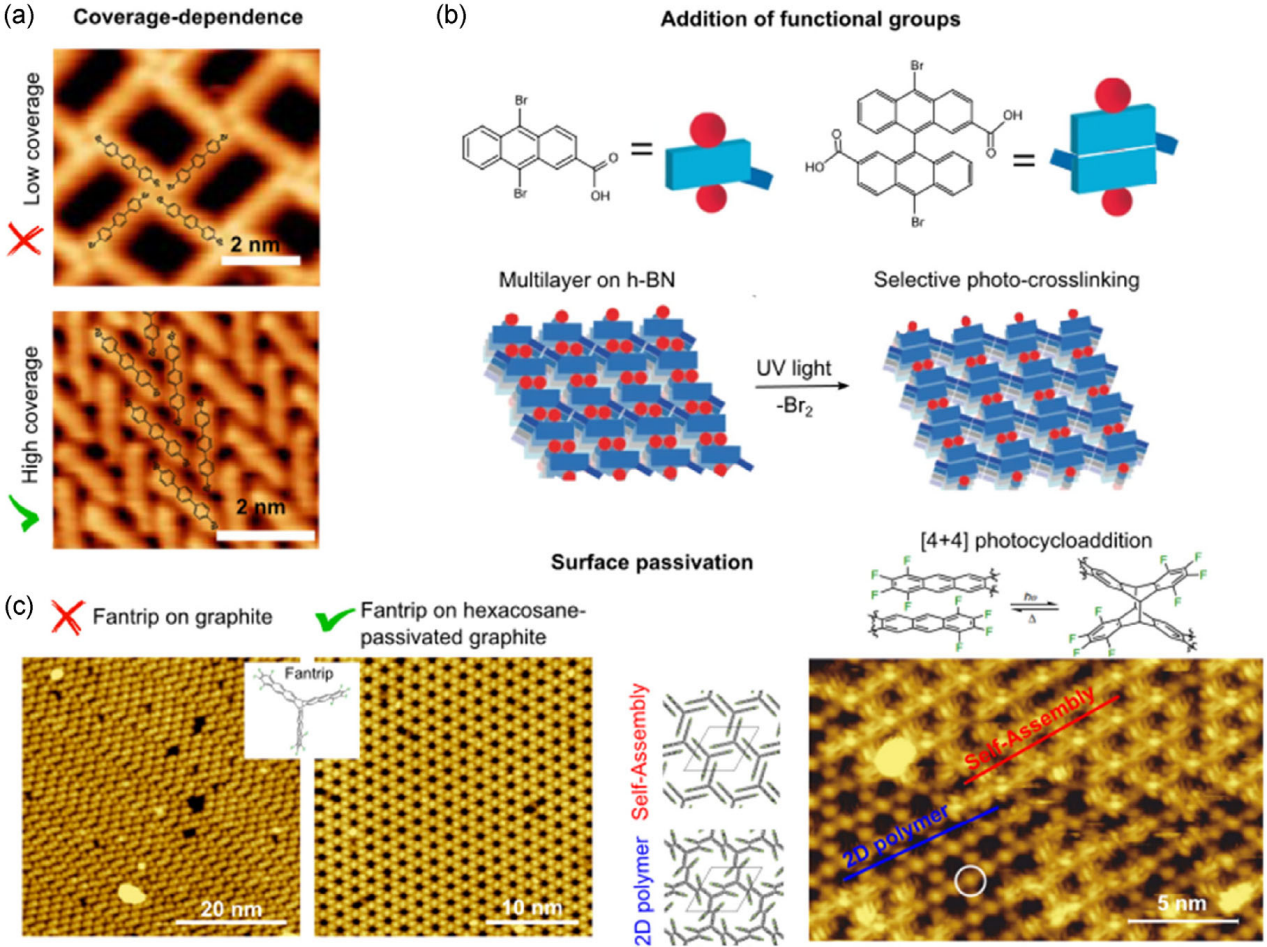
a) Photochemical reaction evolution based on the coverage‐dependent self‐assembly of DBTP on Au(111). STM images Reproduced with permission.^[^ ^39^ ^]^ Copyright (2023), ACS. b) Scheme representing the formation of a self‐assembly driven by the functionalization of the molecular precursor with a carboxylic group, which dictates the selective photo‐crosslinking reaction on hexagonal boron nitride (h‐BN). Reproduced with permission.^[^ ^70^ ^]^ Copyright (2022), Springer Nature. c) [4 + 4] photocycloaddition polymerization of “Fantrip” precursor on hexacosane‐passivated graphite. The formation of the optimal molecular self‐assembly on the passivated surface allows the successful evolution of the photochemical reaction. STM images Reproduced with permission.^[^ ^83^ ^]^ Copyright (2021), Springer Nature Limited.

Nowadays, the most explored on‐surface reactions are thermally activated dehalogenations to induce C—C couplings. However, in the context of light‐induced on‐surface reactions, there are only a few examples. Light induces the first reaction step, dehalogenation. There is evidence of molecular coverage effect in controlling the reaction yield, due to the proper initial molecular rearrangement (see coverage dependance of the self‐assembly in **Figure** **3**a) to facilitate the intermolecular reaction with minimal motion.^[^ ^39^ ^]^ Furthermore, to induce the formation of the right self‐assembly, the functionalization of the molecular precursor with functional groups that drive the intermolecular interactions can be employed.^[^ ^70^ ^]^ In this case, the formation of self‐assembly dictates a selective photo‐crosslinking reaction between the monomers (Figure 3b).

**Figure 4:**
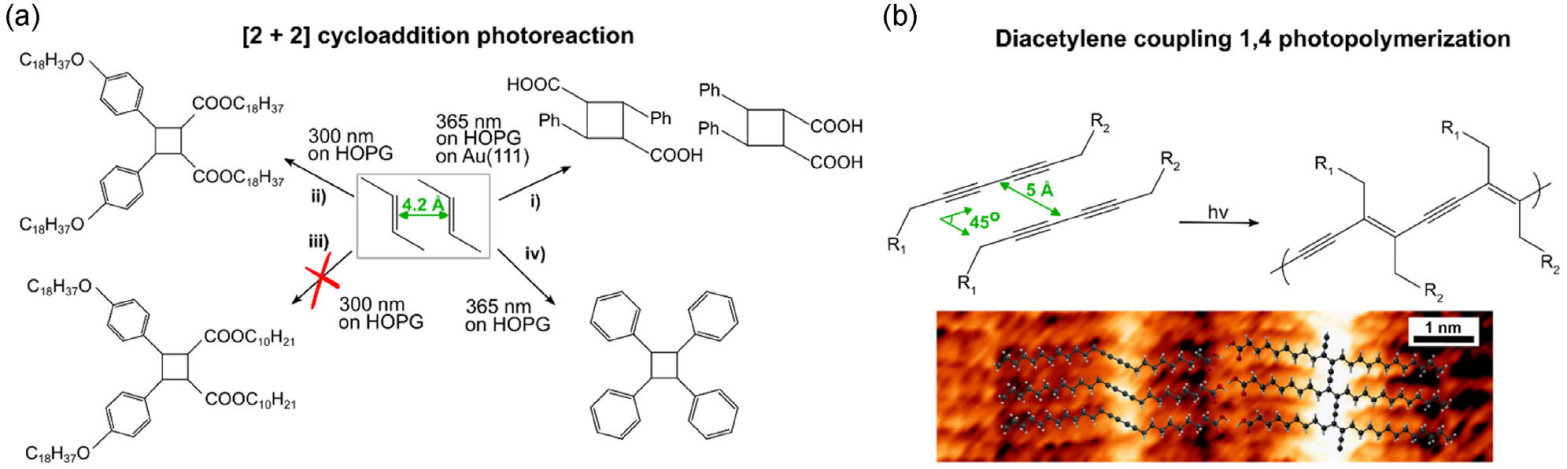
a) Chemical scheme of the [2 + 2] photocycloaddition reaction, illustrating the optimal distances for the reaction evolution according to topochemistry postulates. i) Route toward the reaction of AOCA precursor on Au(111)^[^ ^74^ ^]^ and HOPG.^[^ ^75^ ^]^ ii) and iii) The selective reaction evolution based on the choice of molecular precursor incorporating different alkyl chains.^[^ ^51^ ^]^ iv) Stilbene photodimerization on HOPG.^[^ ^76^ ^]^ b) Reaction scheme of diacetylene coupling 1,4 photopolymerization, illustrating the optimal distances between diacetylene for the reaction to proceed. STM image. Reproduced with permission.^[^ ^80^ ^]^ Copyright (2012), ACS.

The photochemistry of alkenes has been widely investigated, such as in photoisomerizations, addition reactions, cycloaddition, photo‐oxidation, or electrocyclic processes, among others.^[^ ^71^ ^]^ On the surface, so far, only the [2 + 2] cycloaddition reaction has been investigated. The [2 + 2] cycloaddition reaction in a polymer or crystal lattice involves strict geometrical requirements. The reaction can only take place if the double bond distances do not exceed 4.2 Å and are parallel aligned, as illustrated in **Figure** **4**a. In this regard, the photodimerization of cinnamic acid is the most studied reaction.^[^ ^72^ ^,^ ^73^ ^]^ The photochemical cyclodimerization of cinnamate derivatives has been investigated on different surfaces, thanks to the optimal preorganization of the molecules. We find two works reporting the photodimerization of 4‐(amyloxy)‐cinnamic acid (AOCA) on both Au(111)^[^ ^74^ ^]^ and HOPG^[^ ^75^ ^]^ (route (i) in Figure 4a). Remarkably, in the case of Au(111), although the self‐assembly does not exactly follow topochemical postulates, the photoexcitation process drives the molecules into the favorable orientation for photodimerization at the liquid‐solid interface. Therefore, although topochemistry allows the understanding of plenty of organic reactions in the solid state, in the context of photochemistry the dynamics associated with the molecular excitation process could preclude/favor reactions that were expected to be allowed/restricted.^[^ ^73^ ^]^ Another work using cinnamate derivatives on graphite/liquid interface shows two cases where the reaction can take place.^[^ ^51^ ^]^ The study of two different molecular precursors demonstrates the viability of the reaction based on the self‐assembly. In the case of the trans‐C18CinnC18 precursor (route (ii) in Figure 4a), geometrical changes taking place upon photoexcitation promote the [2 + 2] cycloaddition reaction. In contrast, for the other cinnamate derivative (C18CinnC10) under study, the topochemical conditions cancel the reaction (route (iii) in Figure 4a). Additionally, we highlight the reaction of a monoalkene precursor:^[^ ^76^ ^]^ using stilbene derivatives on HOPG, they observe first the photoisomerization that induces the right configuration to subsequently induce the [2 + 2] photodimerization (route (iv) in Figure 4a).

As an extension of alkenes photochemistry, we review the case of the alkynes, whose photochemical activity can be rationalized in a similar way to the alkenes, although the rigid bond cancels some photoreactions and photoisomerizations.^[^ ^71^ ^]^ Furthermore, alkynes in the excited state react very rapidly with protons. Thus, control over their reactivity is more challenging than alkenes. In this context, photochemistry on surfaces under UHV conditions provides an inert environment and geometry conditions, which could be ideal to control the reaction. Terminal alkynes homocoupling has been investigated on Au(111) and Ag(111).^[^ ^77^ ^]^ In this case, strong molecule‐surface interactions on the Au(111) cancel the reaction, thus demonstrating certain limitations to take into account when working on (metallic) surfaces. On the other hand, on Ag(111), the same precursor forms a more packed self‐assembly, suggesting lower interaction with the surface with respect to the Au(111). The photochemical Glaser‐type coupling dimerization occurred within the self‐assembly and at the edge of the islands after UV irradiation. Note that the authors proposed the photon‐induced desorption to play a crucial role for the successful C—C coupling, creating the space needed for proper orientation of the molecular precursor. In this regard, the Glaser‐type coupling photoreaction has been also investigated on nonmetallic surfaces. In another work,^[^ ^43^ ^]^ UV illumination resulted in the homocoupling of terminal alkynes on HOPG in a liquid–solid interface. The molecular precursor is equipped with carboxylic acid units that drive the self‐assembly formation, where neighboring alkynes are properly oriented to react upon illumination and form butadiynyl derivatives. Thus, the photochemical reaction occurs within the self‐assembly. Additionally, the same authors investigated methyl‐acetylene compounds (—C≡C—CH_3_) on HOPG under similar conditions,^[^ ^78^ ^]^ demonstrating a photochemically controlled recombination reaction. UV illumination triggers the photolysis of the precursor generating propargyl radicals that undergo C—C coupling reaction leading to two possible outcomes: the formation of dimers (and a few oligomers) connected by 1,5‐hexadiyne (—C≡C—CH_2_—CH_2_—C≡C—) or para‐phenylene (—C_6_H_4_—) groups.

Diynes, such as diacetylenes, have also been explored on graphitic surfaces. Topochemical conditions have been deeply explored for diacetylenes, where so far, the optimal conditions are those presenting a stacking distance between diacetylenes of 5 Å and 45° (see scheme in Figure 4b).^[^ ^79^ ^]^ On surfaces, the deposition of 10,12‐pentacosadiynoic acid molecules on epitaxial graphene grown on a SiC(0001) substrate and under UHV conditions, results in the formation of a self‐assembly tracking the morphology of the graphene.^[^ ^80^ ^]^ After UV illumination, the diacetylene units of adjacent molecules react following the photopolymerization reaction shown in Figure 4b. Other diacetylenes precursors undergoing a similar 1,4 photopolymerization reaction have been explored, such as the isophthalic acid derivative (ISA‐DIA) and terephthalic acid derivative (TTA‐DIA) at the air‐HOPG interface.^[^ ^81^ ^]^ The initial orientation of the diacetylene units is the key to the successful evolution of the reaction for both molecular precursors: the stacking distance and the angle between stacking axis and the diacetylene is 4.8 Å and 50° for ISA‐DIA, and 4.85 Å and 47° for TTA‐DIA. As illustrated in Figure 4b, these values are close to the optimal parameters for maximal reactivity. The density of polymerized ISA‐DIA chains per unit area is twice that of TTA‐DIA, probably due to the rigidity of the latter one. Additionally, experiments of ISA‐DIA under liquid‐graphite interface confirm higher degree of polymerization,^[^ ^82^ ^]^ tentatively assigned to a higher mobility of the molecules under these conditions. These results highlight the importance of considering additional factors, including molecular design and reaction conditions, in optimizing topochemical‐promoted reactions.

In the context of OSS, the study of aromatic compounds has attracted most of the attention. However, the study of their photoactivity on surface is, so far, limited with respect to thermal‐induced reactions. The two main examples consist of [4 + 4] photocycloaddition: dimerization^[^ ^54^ ^]^ and polymerization.^[^ ^83^ ^]^ Although the reaction is “similar”, two different strategies are employed to induce the optimal preorganization: in the first case, they impose a favorable geometry for the C—C coupling reaction by adding S‐anchoring groups that form Au‐thiolate bonds with the surface^[^ ^54^ ^]^ (Figure 2d); while in the second case it was found that surface passivation of graphite by alkane hexacosane reduces the π–π interactions between the anthracene moieties and the graphite to favor the formation of the right self‐assembly^[^ ^83^ ^]^ (Figure 3c). The [4 + 4] photocycloaddition reaction has been deeply investigated under different conditions. It presents a very high yield (>90%) and was one of the first photoreactions reported for aromatic compounds, thus highlighting the relevance of this reaction also in the context of on‐surface photochemistry. Finally, the [2 + 2] photocycloaddition of fullerenes has been reported using calcite as substrate.^[^ ^84^ ^]^ This approach is particularly interesting because the substrate periodicity along a direction matches the distances of linked fullerenes, resulting in a selective C—C coupling reaction along a specific direction upon illumination. This work highlights that the surface‐template effect can facilitate the photoreaction evolution. In this regard, another relevant example focused on the photoactivity of dimaleimide on the KCl substrate:^[^ ^85^ ^]^ upon UV illumination, the photoinitiated radical polymerization follows a preferred growth axis with respect to the surface, driven by the interaction between the potassium cations at the surface and oxygen atoms from the molecular precursor.

Although the formation of packed assemblies on a 2D surface imposes certain configurations and the motion of the molecules is restricted under cryogenic conditions, these works demonstrate different strategies to modulate the formation of the self‐assembly. Among other strategies, the choice of the substrate, the molecular coverage, passivation strategies, and the addition of anchoring groups allowed the investigation of photochemical reactions deeply investigated in solution or organic crystals and, in some cases, to overcome their limitations. According to topochemistry postulates, the solid‐state packing of organic crystals can direct their reactivity. Therefore, the possibility of controlling molecular preorganization and the inert conditions provide unique opportunities for the study of new photochemical reactions.

## 5 Stabilization of Reactive Species

The ability to generate and stabilize reactive chemical species is crucial for understanding reaction mechanisms and developing new synthetic strategies. While many reactive intermediates are too short‐lived to be isolated under conventional conditions, surfaces provide a suitable environment for stabilizing these elusive species. One important parameter is the possibility of inducing chemical reactions at low temperatures, where these species exhibit lower reactivity and the surface diffusion is limited.

**Figure 5:**
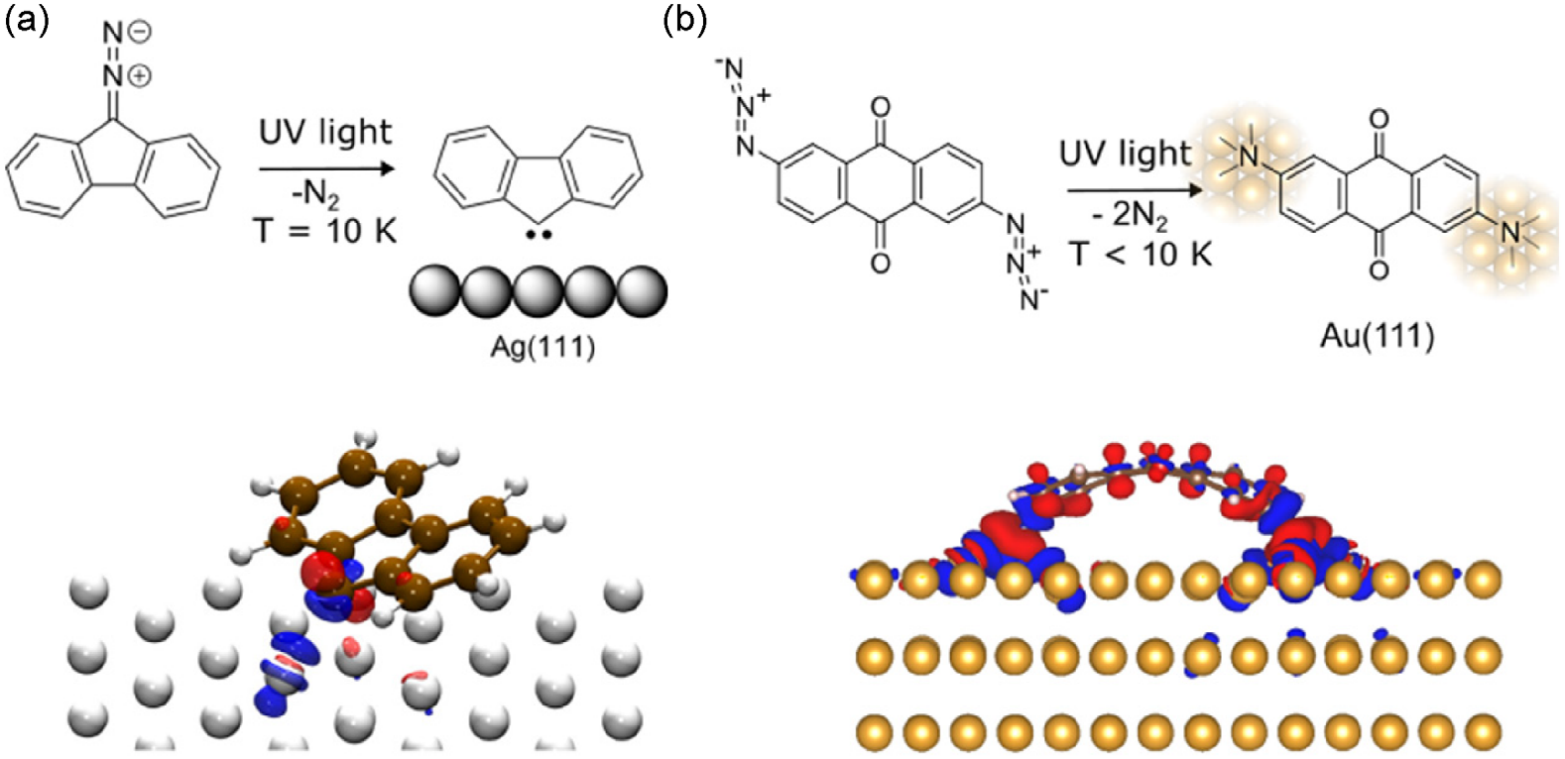
a) Carbene intermediate resulting from the photodissociation of 9‐diazofluorene on Ag(111) (top) and difference in electron density between the molecule adsorbed on metal and the individual components at the same atomic positions (bottom). Reproduced with permission.^[^ ^86^ ^]^ Copyright (2021), ACS. b) Dinitrene intermediate resulting from the photodissociation of 2,6‐diazidoanthracene‐9,10‐dione on Au(111) (top) and computed charge transfer (bottom). Reproduced with permission.^[^ ^49^ ^]^ Copyright (2025), Wiley. In both bottom panels, blue and red densities represent charge depletion and accumulation, respectively.

Surface‐stabilized carbene and nitrenes, the most widely studied intermediates in organic chemistry, can be generated by following a similar procedure, i.e., by N_2_ photolysis from diazo and azido compounds, respectively. The photodissociation of N_2_ using 9‐diazofluorene precursor on Ag(111) leads to the formation of fluorenylidene (**Figure** **5**a), stabilized thanks to the strong interactions between the carbene and the surface (see calculated structure of fluorenylidene on Ag(111) at the bottom panel of Figure 5a) at cryogenic temperatures.^[^ ^86^ ^]^ Another example of carbene isolation was reported using methyl diazoacetate as precursor:^[^ ^58^ ^]^ after irradiation, activation, and N_2_ release, a metastable Ag‐stabilized methyl ester‐carbene is generated. With respect to nitrenes, an aryl azide precursor was employed to generate a copper–nitrene intermediate.^[^ ^87^ ^]^ This intermediate was spectroscopically detected, and it was shown to undergo further ISC from the singlet to the triplet nitrene, followed by the dimerization reaction forming an azo (N=N) bond.^[^ ^87^ ^]^ This approach was extended to dinitrenes using a diazide precursor (see Scheme in Figure 5b).^[^ ^49^ ^]^ In this case, the strong interaction between the dinitrene and the Au(111) surface (calculated charge transfer between the dinitrine and Au(111) surface is illustrated at the bottom panel Figure 5b) allowed the visualization of such compounds by STM and nc‐AFM, although it precludes further reactions in the absence of thermal activation.

The stabilization of species by molecule‐surface interactions is closely related to surface functionalization experiments, allowing the selective introduction of defects, changes in the work function, and tuning the reactivity of the surface. The photodecomposition of benzoyl peroxide on graphene allowed the formation of a phenyl radical that attacks the graphene substrate, creating sp^3^ carbon “defects”.^[^ ^88^ ^]^ In this context, other works explore the functionalization of graphene by light, using photochemically generated phenazine radicals^[^ ^89^ ^]^ or the selective photocycloaddition reaction.^[^ ^90^ ^]^ Other surface functionalization strategies have been investigated, such as employing TiO_2_, where the oxygen adatoms from the surface, combined with the UV light, induce the photofragmentation of butyrophenone, resulting in the release of propyl radical while the benzoate remains bonded to selective Ti atoms of the surface.^[^ ^50^ ^]^

## 6 Summary and Outlook

The field of on‐surface photochemistry is still in its infancy, and the understanding of well‐established concepts from photochemistry can promote its advancement. Still, when working on a surface, there are some specific aspects that must be considered, such as the photoexcitation process, the imposition of certain conformations of molecular precursors, or the charge transfer between the surface and molecules, either in the ground or excited states. The progress in on‐surface photochemistry depends strongly on the ability to prepare preorganized molecular precursors, which are photochemically active. Therefore, there is a quest for concerted action of organic synthesis to design and synthesize suitable candidates and surface science to devise different strategies to impose an optimal molecular rearrangement to control the reaction outcomes. Understanding reaction mechanisms is crucial for designing successful experiments. In this sense, the development of photoreactivity on insulating surfaces opens the possibility to investigate fundamental mechanistic aspects on single molecules, differently than metallic surfaces where both direct and indirect (i.e. metal‐mediated photogenerated charge transfer) mechanisms occur and are hard to disentangle. Hence, photoreactions on insulating surfaces are ideal systems to be investigated both experimentally and theoretically.

The future of the field could be closely related to the great advances in STML^[^ ^37^ ^,^ ^91^ ^]^ and tip‐enhanced Raman spectroscopy (TERS),^[^ ^92^ ^,^ ^93^ ^]^ achieving sub‐molecular resolution and understanding the photophysical properties of different molecules. The next natural step is to exploit plasmonic catalytic nanocavities to induce photochemical reactions at the single‐molecule level. In this regard, there are only a limited number of works reported.^[^ ^94^, ^95^, ^96^, ^97^, ^98^ ^–^ ^99^ ^]^ Nevertheless, the possibility to get experimental knowledge about the mechanism of plasmon‐induced chemical reactions has been demonstrated.^[^ ^95^ ^]^ This strategy can contribute to expanding the photochemical reactions triggered by visible light feasible on the surface with sub‐molecular control.

## Conflict of Interest

The authors declare no conflict of interest.
